# Role for migratory domestic poultry and/or wild birds in the global spread of avian influenza?

**DOI:** 10.1080/01652176.2019.1697013

**Published:** 2019-11-22

**Authors:** Johannes H. van der Kolk

**Affiliations:** Swiss Institute for Equine Medicine (ISME), Vetsuisse Faculty, University of Bern, Bern, Switzerland

Recently, the Dutch bird migration atlas has been launched (https://vogeltrekatlas.nl) based on more than 13 million ring recovery data obtained during the previous 100 years. As such it is a very valuable source on information regarding bird migration.

Within veterinary medicine bird migration is nowadays frequently associated with avian influenza (AI). However, AI virus strains have been circulating and diversifying in wild bird populations for at least the last 100 years (Lycett et al. 2019). AI viruses - in particular highly pathogenic avian influenza (HPAI) [or previously called ‘fowl plague’] viruses - form a continuous threat to the poultry industry, public health, and to some wild bird species (van den Brand et al. 2018). For instance, it has been estimated that up to 11–39% of the wintering population of peregrine falcons (*Falco peregrinus*) in the Netherlands might have died due to HPAI A/H5N8 virus strains during autumn–winter 2016–2017 (Kleyheeg et al. 2017). Sporadic infections of humans with a limited number of avian virus subtypes (H5, H6, H7, H9, H10) have been known to occur directly from avian sources, but without as yet leading to sustained human to human transmission (Yuen et al. 1998, Koopmans et al. 2004, Fouchier et al. 2004, Shi et al. 2013, Parry 2013, Chen et al. 2014, Bui et al. 2017, Lycett et al. 2019). Typically, these infections are severe in humans, often causing death (Lycett et al. 2019) including the regrettable death of a veterinarian from pneumonia after infection with AI A/H7N7 virus. The 57-year-old veterinarian became ill within days of visiting a poultry farm hit by an AI outbreak (Sheldon 2003). It has been stated that direct transmission of the virus from wild birds to humans appears to be very rare (or non-existent), presumably due to the low frequency of contact between the two populations. However, transmission from domestic avian species to humans does occur, especially in live bird markets in Asia (Lycett et al. 2019).

Of course, it is important not to confuse the threat posed by HPAI with that of a human flu pandemic (Sheldon 2005). In 1918, a H1N1 strain of influenza A virus, the ‘Spanish flu’, caused a human pandemic resulting in the deaths of 50 million people. Since then, three other human influenza A virus pandemics have occurred: H2N2 in 1957 (‘Asian flu’), H3N2 in 1968 (‘Hong Kong flu’), and H1N1 again in 2009 (‘swine flu’)(Lycett et al. 2019). It has been suggested that the human H2N2 and H3N2 pandemic viruses might have had an avian origin on the basis of antigenic cross-reactivity (Pereira et al. 1967). However, the subsequent two human pandemics (H2N2 in 1957 and H3N2 in 1968) were not caused by completely avian-origin viruses, but were rather reassortant viruses with avian-origin HA, PB1 polymerase and (for the 1957 pandemic) NA segments (Kawaoka et al. 1989, Bean et al. 1992, Schäfer et al. 1993, Joseph et al. 2015, Lycett et al. 2019). The 2009 H1N1 ‘swine flu’ pandemic was a result of reassortment between different strains of influenza A virus that had been circulating in swine for at least 10 years (Smith et al. 2009, Lycett et al. 2019).

Since the emergence of the HPAI A/H5N1 virus in poultry in China in 1996, H5 HPAI viruses that share a common ancestral virus strain (A/goose/Guangdong/1/96 [GsGd]) have continued to cause outbreaks in poultry (Duan et al. 2008). The hemagglutinin (H) gene of the HPAI A/H5N1 virus diversified into multiple genetic lineages (“clades”). More recently, reassortment between the HPAI A/H5N1 virus and the low pathogenic avian influenza (LPAI) viruses resulted in HPAI viruses with neuraminidase (N) genes (N1, N2, N5, N6, and N8) and other genes of LPAI virus origin (Zhao et al. 2012, Liu et al. 2013, Zhao et al. 2013, Wong et al. 2015). From China, H5 GsGd virus has been introduced to other Asian countries, the Middle East, Africa, and Europe. Within Europe, HPAI A/H5N1 GsGd virus has been detected in multiple countries in 2004 (clade 1), 2005/2006/2007 (clades 2.2 and 2.2.1), and 2008/2009/2010 (clade 2.3.2)(Cattoli et al. 2009, Reid et al. 2011). In November and December of 2014, the HPAI A/H5N8 GsGd virus (clade 2.3.4.4, group A, Buan-like)(Jeong et al. 2014) was detected in various countries of Asia, Europe, and - for the first time - North America (van den Brand et al. 2018). HPAI A/H5N6 virus as a novel reassortant of the H5N8 clade 2.3.4.4 group B viruses (Beerens et al. 2018) was first detected at the Russia - Mongolia border in May 2016 (Beerens et al. 2019). Remarkably, the first detection of HPAI A/H5N6 virus in the Netherlands as well as in Europe (Beerens et al. 2018) was on a commercial farm notably keeping meat ducks no sooner than on December 7, 2017 (Beerens et al. 2019).

HPAI is essentially a poultry disease (van den Brand et al. 2018). The intranasal inoculation with A/chicken/Hong Kong/220/97 (A/H5N1) influenza virus in chicken was lethal within 1.5-2 days post inoculation (Perkins and Swayne 2001). Conversely, in ducks the A/H5N1 Hong Kong isolates produced innocuous infection characterized by transient shedding and no clinical disease. This limited (subclinical) infection is typical of AI in this subfamily of waterfowl (Easterday et al. 1997, Perkins and Swayne 2001). Vaccination in chicken has been characterized as a logistically demanding and costly method with inherent uncertainties under field conditions regarding the level and duration of protection against infection (OIE/FAO/IZSVe Scientific Conference 2007). However, the apparent success of the H7N9 vaccination program in China suggests that it is possible to control virus circulation in domestic birds and thus vastly reduce the number of human infections and the risk of ongoing human to human spread (Lycett et al. 2019).

**Figure 1.**
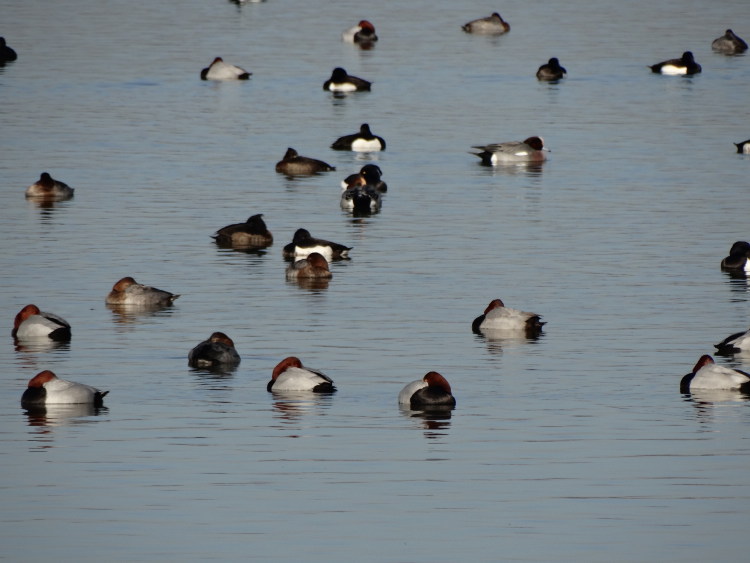
Two wintering male Eurasian wigeons (Anas penelope) (with a yellowish forehead) among tufted ducks (Aythya fuligula) and Eurasian pochards (A. ferina) on 15 February, 2017 at Nuldernauw, Putten, the Netherlands (photograph by the author).

**Figure 2.**
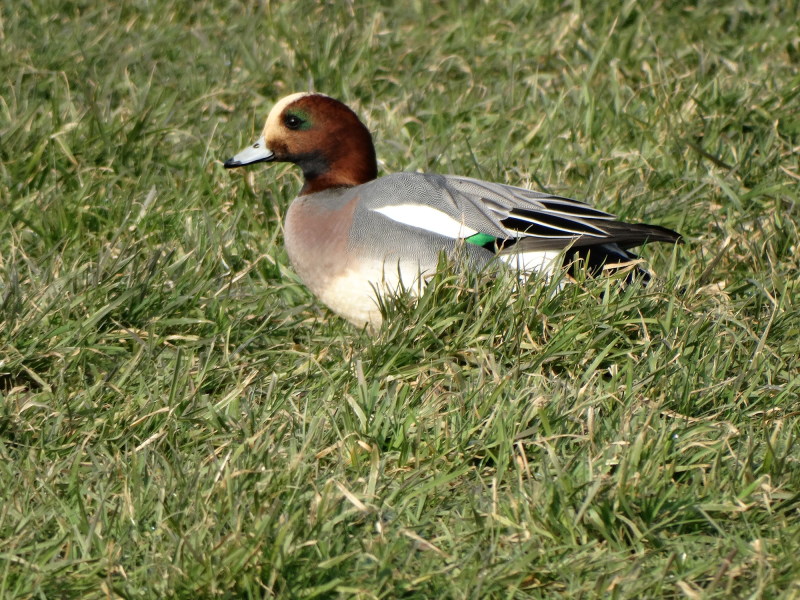
Wintering male Eurasian wigeon (Anas penelope) on 21 February, 2018 at Polder Arkemheen, Nijkerk, the Netherlands (photograph by the author). Over the years 2013-2017 on the average 665,200 Eurasian Wigeons wintered in the Netherlands (Hornman and van Winden 2018) .

AI was also studied in captive ducks of six different species, namely tufted duck (*Aythya fuligula*), Eurasian pochard (*A. ferina*), mallard (*Anas platyrhynchos*), common teal (*A. crecca*), Eurasian wigeon (*A. penelope*), and gadwall (*A. strepera*). Birds were 8–11 months of age at time of inoculation and captive-bred. Prior to simultaneous tracheal and esophageal inoculation with HPAI A/H5N1 virus some animals had positive or suspected positive antinucleoprotein antibody titres. Inoculation with the 2005 (A/H5N1) virus caused clinical signs at 3–4 days following inoculation in both diving duck species, which were more severe in tufted ducks than in Eurasian pochards. Viral excretion was highest in Eurasian pochards and mallards. Severely affected birds died or were euthanized in a moribund state at 4 days post inoculation (Keawcharoen et al. 2008). A similar study was performed in captive ducks of the species Eurasian wigeon (Figures 1 and 2), common teal, mallard, and Eurasian pochard. The birds were approximately one year of age and also captive-bred. Prior to simultaneous tracheal and esophageal inoculation with HPAI A/H5N8 virus some animals were RT-PCR positive for the matrix gene of influenza A virus. Inoculation with the 2014 (A/H5N8) virus was subclinical in all 4 duck species and virus excretion was highest in Eurasian wigeons (van den Brand et al. 2018). While the HPAI Asian A/H5N1 viruses are 100% lethal for chickens and other gallinaceous poultry, the absence of disease signs in some duck species has led to the concept that ducks are the “Trojan horses” of A/H5N1 in their surreptitious spread of virus (Kim *et al* 2009). The captive ducks in both inoculation studies were obtained from two different breeders. The presence of some captive-bred birds with either (suspected) positive antinucleoprotein antibody titres or RT-PCR positivity for the matrix gene of influenza A virus might illustrate this concept.

Fortunately, influenza viruses - including AI - do not result in persistent viral infection. For instance, HPAI A/H5N1 virus does not persist in individual domestic ducks after acute infection (Wibawa et al. 2014). In line, it has been shown that the 2014 HPAI A/H5N8 virus has not continued to circulate extensively in wild bird populations since the winter 2014–2015 in the Netherlands and that independent maintenance of the virus in these populations appears unlikely (Poen et al. 2018). Of note, there were no significant differences between chicken and ducks in the duration of HPAI virus shedding *via* both respiratory [2.6 (1.1–6.5) versus 6.9 (2.8–17.1) days] or cloaca routes [2.5 (1.0–6.2) and 6.6 (2.7–16.3) days] (Germeraad et al. 2019).

The long-distance spread of HPAI H5 GsGd virus from Asia to other continents is thought to occur *via* migratory birds or poultry trade (van den Brand et al. 2018). Recently, a consortium advocated a role for migratory wild birds in the global spread of AI A/H5N8 virus (the Global Consortium for H5N8 and related influenza viruses 2016). The goal of their study was to analyze the available genetic, epidemiological, and ornithological data for evidence of the relative contributions from poultry trade and from wild bird movements for the global spread of HPAI A/H5N8 clade 2.3.4.4 virus during 2014–2015. In order to address the contribution of poultry trade, data from the Food and Agriculture Organization of the United Nations (FAO) for 2011 to 2013 only on export and import of live domestic ducks and chickens of affected countries were reviewed to estimate the risk of spread of HPAI virus. The publication goes along with a table showing data on the import and export of live poultry in those affected countries without indicating the countries of destination of the exported live animals. South Korea reported the official export of about 3000 live chickens in 2013, whereas China exported more than 3 million live chicken in 2013 without any official export of live ducks over the years 2011-2013. Although the consortium considered that unreported cross-border trade cannot be excluded they concluded - based on this table only - that it seems unlikely that international trade in live poultry played a major role in the long-distance spread of South Korean clade HPAI A/H5N8 virus in 2014–2015 which seems somewhat narrow based and not fully in perspective. In contrast, it has been reported, for example, that chickens transported 1500 km from Lanzhou, Gansu Province, China, apparently introduced H5N1 to Lhasa, Tibet, in January 2004 (FAO 2004, Melville and Shortridge 2004, Greger 2006). Furthermore, every day, millions of live poultry are moved around the world by ground, air and sea transport, which potentially could carry for instance H5N1 to fresh areas (Butler and Ruttiman 2006). In line, during October and November 2005, customs inspectors at the US Department of Agriculture (USDA) at a port in California intercepted nearly 75 tonnes of poultry smuggled in from Asia (Butler and Ruttiman 2006). Also, the first outbreak of H5N1 in Africa - in Nigeria - was widely attributed to migratory birds. But many now see the imports of day-old chicks as a more plausible cause (Butler and Ruttiman 2006, Greger 2006). Last but not least, remarkably a H5N1 influenza A virus was isolated from duck breast meat processed for human consumption imported to Japan from Shandong Province, China in 2003. Of note, this duck meat isolate was highly pathogenic to chickens upon intravenous or intranasal inoculation, replicated well in the lungs of mice and spread to the brain, but was not as pathogenic in mice as H5N1 human isolates (Mase et al. 2005).

**Figure 3.**
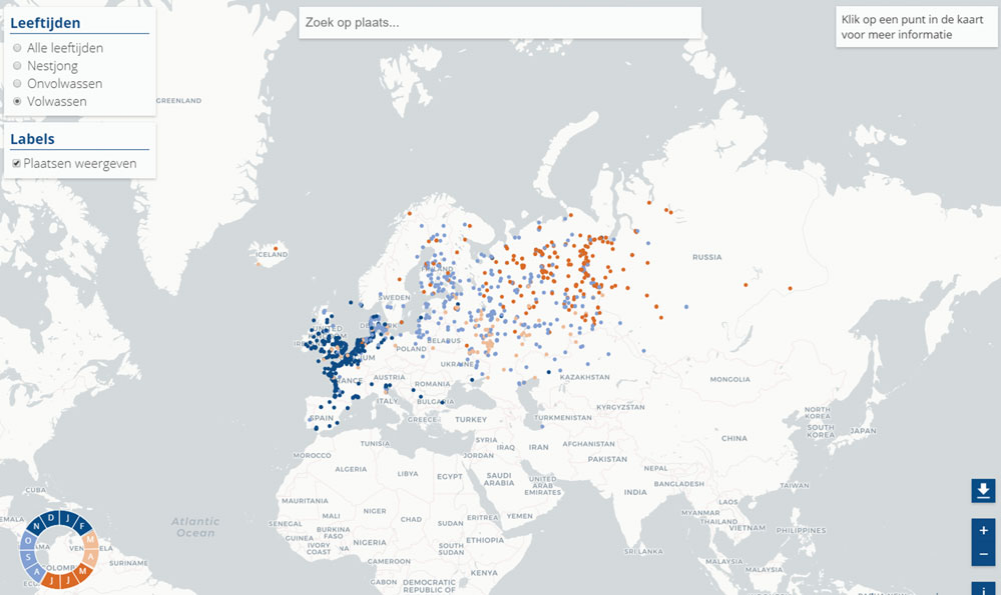
Ring recovery data of adult Eurasian wigeon (Anas penelope) with the colour of the dot indicating the month of recovery as illustrated by the circle in the lower left part of the map (map reproduced with permission of het Vogeltrekstation, Wageningen, the Netherlands).

Migratory birds like the Eurasian wigeon have a distinct flyway (Figure 3), which is by far less bizarre than flyways of poultry meat and meat products (including live birds) as illustrated in the paper by Ventura da Silva (2013). Despite wild bird migration HPAI virus - for the first time – managed in the end to reach North America as A/H5N8 (van den Brand et al. 2018). Of note, HPAI A/H5N6 virus was not detected in wild birds after March 2018, but in August 2018 infected wild birds were found again in the Netherlands (Beerens et al. 2019). These observations seem to be in line with the statement that the geographic spread of the disease does not correlate with migratory routes and seasons, but that the pattern of outbreaks follows major road and rail routes, not flyways (Editorial 2006). In accord, it has been stated that migratory geese are exposed to the virus after their arrival on wintering grounds, indicating that migratory geese might not disperse low pathogenic avian influenza virus during autumn migration (Yin et al. 2017).

**Figure 4.**
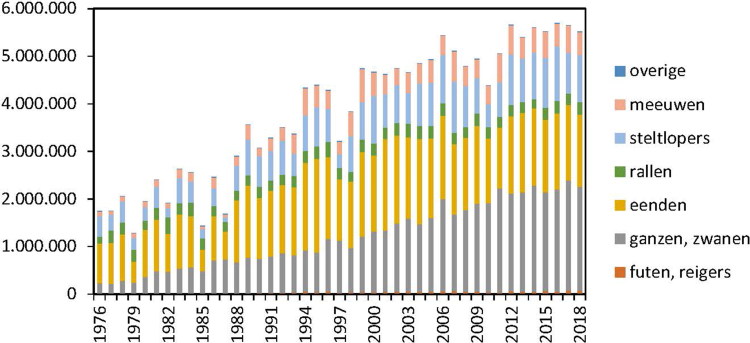
Numbers of wintering birds in the Netherlands from 1976-2018 comprising geese and swans (gray), ducks (yellow), rails (green), waders (light blue), gulls (pink), grebes and herons (brown) and others (dark blue) (figure reproduced with permission of SOVON, Nijmegen, the Netherlands).

Wintering in the Netherlands is increasingly popular among wild birds (Figure 4) like the Eurasian wigeon as an average 665,200 birds over the years 2013–2017 prefer to stay there in winter time (Hornman and van Winden 2018). Due to additional migration of other wild duck species over the years 2013–2017 on the average more than 1.5 million wild ducks in total winter in the Netherlands (Figure 4 ducks in yellow bars). These birds add to the around 100 million chickens and about 0.9 million domestic ducks also living in the Netherlands (van der Peet et al. 2018). Last but not least, the migration of live domestic ducks into the Netherlands in 2013 counted 3.7 million birds whereas outward migration of live domestic ducks counted 1.2 million birds (the Global Consortium for H5N8 and related influenza viruses 2016). As such the migration of domestic ducks into the Netherlands by far outnumbers the migration of wild ducks into the Netherlands. It is likely that the ongoing passive wild bird surveillance programme in the Netherlands (Beerens et al. 2019) is by-passed by these domestic ducks. Of note, an ongoing AI surveillance programme monitors farms keeping meat ducks and broiler chicken once yearly *via* serology.

In modern industrial poultry farms without a free-range system, close contact with wild birds is unlikely and strict biosecurity measures are in place to reduce most indirect transmission routes. It was therefore remarkable that outbreaks of HPAI A/H5N8 in Germany, the Netherlands and the United Kingdom in 2014–2015 occurred on modern farms with indoor poultry housing and that no outdoor production sites were affected (European Food Safety Authority (EFSA) 2014, Velkers et al. 2017).

In the six months before the first detection on 14 November 2014 of HPAI A/H5N8 in poultry in the Netherlands, a total of 2,745 wild birds belonging to the orders Anseriformes and Charadriiformes had been sampled for HPAI H5 virus during the wild bird surveillance programme compared to 3,698 birds in the three months afterwards. Remarkably, in the six months before the first detection of HPAI A/H5N8 in poultry sampling was always negative, whereas sampling for virus revealed two European wigeons (out of 1,185) positive for HPAI after the first detection of HPAI H5N8 (Verhagen et al. 2015). Similarly, first detection in the Netherlands of HPAI A/H5N6 was on a commercial farm with meat ducks on 7 December, 2017, whereas the virus was first detected in wild birds [mute swan (*Cygnus olor*)] on 9 December 2017 (Beerens et al. 2019). The most likely conclusion based on these two studies seems that the outbreak in poultry preceded the outbreak in wild birds.

A similar high pathogenicity was measured for H5N6 and H5N8 group B viruses in 6-week-old domestic Pekin ducks. After intravenous inoculation with the H5N6 virus from 2017 all 10 ducks died on the first day, whereas regarding the H5N8 2016 virus, nine ducks died on day 1, and one duck on day 2 (Beerens et al. 2019). Given the very high pathogenicity shown for these H5N6 and H5N8 viruses it cannot be excluded that the outbreak in wild birds in the previous paragraph refers to an inevitable side-effect of the culling process as there are no known natural reservoirs of HPAI (Swayne and Suarez 2000). On the other hand, such high levels of pathogenicity in wild birds might severely limit their role in the movement of virus (Melville and Shortridge 2004). Furthermore, wild birds infected with HPAI A/H5N6 virus related to the first outbreak in poultry were found at short distances from the farm, within a short time frame, whereas no wild bird viruses related to outbreaks 2 and 3 were detected in winter 2017–2018 (Beerens et al. 2019). In winter 2016–2017, HPAI A/H5N8 virus amplification primarily took place within the Netherlands resulting in associated die-offs of at least 57 wild birds belonging to 12 species (Poen et al. 2018).

Experts think human influenza started about 4,500 years ago with the domestication of waterfowl like ducks, the original source of all influenza viruses. Farmers moved ducks from the rivers and tributaries onto flooded rice fields to be used as an adjunct to rice farming. This led to a permanent year-round gene pool of avian influenza viruses in East Asia in close proximity to humans (Shortridge 2003, Greger 2006) or in other words this region "represents the most incredible reassortment laboratory for influenza viruses that anyone could ever imagine" (Das 2002, Greger 2006). The domestic duck of southern China is now considered the principal host of all influenza viruses with pandemic potential (Shortridge 1992, Greger 2006). As mentioned before, because HPAI does not necessarily kill its anseriform hosts, reassortment with co-circulating LPAI viruses can occur, furthering evolution of the virus (Lycett et al. 2019). Median‐joining network analysis was performed based on the genomes of HPAI A/H5N6 viruses isolated from three commercial poultry farms (comprising two duck and one chicken farm) in the winter 2017-2018 revealing several amino acid changes (Beerens et al. 2019). However, when using this technique under field conditions over a period of more than 90 days it seems prudent to consider the species involved as well as the maximal life-span of about 7 weeks regarding meat ducks and broiler chicken as the process of viral reassortment might be more typical for ducks than chicken.

Regarding the role for migratory domestic poultry and/or wild birds in the global spread of avian influenza the impact of domestic poultry seems still most important. Furthermore, the risk has been highlighted of the live bird trade which could be one of the possible routes for the introduction and dissemination of both LPAIV and HPAIV throughout the world (Lee et al. 2019). Far more likely to be perpetuating the spread of the virus is the movement of poultry, poultry products, or infected material from poultry farms - e.g. animal feed and manure (Editorial 2006). Contaminated food and water, animal/insect vectors and air can play a role in the secondary spread of AI virus within and between poultry flocks, but movement of man and fomites is considered most relevant for spread between farms (Alexander 2007, Velkers et al. 2017). In line, it has been suggested that between-farm transmission contributes considerably to the incidence of LPAI virus infections in poultry (Bergervoet et al. 2019).

In conclusion, previous editorials entitled ‘Influenza: time to come to grips with the avian dimension’ (Melville and Shortridge 2004) and ‘Avian influenza goes global, but don't blame the birds’ (Editorial 2006) still hold. Hence increasing biosecurity and vaccination in domestic poultry are likely to be important strategies to keep outbreaks in these populations to a minimum (Lycett et al. 2019). With reference to vaccination in domestic poultry domestic ducks seem to be of utmost importance.

Johannes H. van der Kolk, Editor-in-Chief *Swiss Institute for Equine Medicine
(ISME), VetsuisseFaculty, University of Bern, Bern, Switzerland*
johannes.vanderkolk@vetsuisse.unibe.ch
